# Antioxidant properties of *allium turcicum* Özhatay & cowley plant extract, its effects on the proliferation and migration of cancer cells

**DOI:** 10.3389/fphar.2024.1438634

**Published:** 2024-09-20

**Authors:** Polat İPEK, Ayse Baran, Deniz Barış Cebe, Elham Ahmadian, Aziz Eftekhari, Mehmet Fırat Baran

**Affiliations:** ^1^ Department of Physiology, Faculty of Veterinary Medicine, Dicle University, Diyarbakir, Türkiye; ^2^ Department of Biology, Graduate Education Institute, Mardin Artuklu University, Mardin, Türkiye; ^3^ Department of Chemistry, Batman University Faculty of Science, Batman, Türkiye; ^4^ Kidney Research Center, Tabriz University of Medical Sciences, Tabriz, Iran; ^5^ Research Center for Pharmaceutical Nanotechnology, Biomedicine Institute, Tabriz University of Medical Sciences, Tabriz, Iran; ^6^ Department of Biochemistry, Faculty of Science, Ege University, Izmir, Türkiye; ^7^ Department of Life Sciences, Engineered Biomaterials Research Center, Khazar University, Baku, Azerbaijan; ^8^ Department of Food Technology, Vocational School of Technical Sciences, Batman University, Batman, Türkiye

**Keywords:** Allium turcicum Özhatay & Cowley, cancer, migration, Cytotoxicity, cell viability, glioblastoma

## Abstract

Cancer is a type of non-communicable disease that is responsible for numerous deaths worldwide. Cancer incidence and mortality rates are on the rise due to a combination of factors, such as a growing population, aging, and poor dietary habits. The *Allium turcicum* Özhatay & Cowley plant is an endemic plant in the area where it grows and is consumed by the public due to its various benefits. This endemic plant, which generally grows in high-altitude regions, is sold in bunches because it is costly, mixed with rock salt, crushed into powder, and consumed as a spice. The cytotoxic and growth-inhibitory effects of *A. turcicum* Özhatay & Cowley herb extract on human glioblastoma U373 cells, human colorectal carcinoma cell HCT-116, and healthy HUVEC cell lines were determined by the MTT method. After 24 and 48 h of application, logIC_50_ values in HUVEC, HCT-116, and U373 cells were defined as 3.737, 3.765; 3.513, 3.696, 4.476, and 4.104 μg/mL, respectively. We conducted a cell migration experiment to study the *A. turcicum* Özhatay & Cowley Extract (ATÖCE) impact on cancer cells’ metastatic behavior. Our findings indicate that ATÖCE has an inhibitory effect on the migration potential of the cells used in the study. We conducted experiments using DPPH, ABTS, CUPRAC, and total phenolic content to assess the antioxidant properties of ATÖCE. The findings from the antioxidant activity experiments revealed an activity level of 0.20 ± 0.046 at IC_50_. Additionally, the total phenolic content was measured to be 0.26 ± 0.044 mg GAE/g.

## Introduction

More than 100 types of cancer worldwide cause approximately 9.6 million deaths annually, and the number of new cases is expected to double in the next 2 decades (Velur and Kusanur, 2022). As a result, there is a pressing need to find new anticancer drugs that operate safely (Torre et al., 2016; Negi and Jain, 2022). Throughout history, plants have been utilized for their medicinal properties in treating cancer (Howes, 2018). More than 49% of current cancer-fighting drugs are derived from natural sources (Li and Vederas, 2009; Newman and Cragg, 2016). Chemotherapy can cause several side effects, including anemia, loss of appetite, bleeding, fatigue, fertility issues, hair loss (alopecia), infection, neutropenia, and lymphedema (Ingole et al., 2024). To avoid these adverse effects, natural plants or their extracted compounds can be an effective alternative to chemical drugs during cancer treatment. These natural remedies have been utilized for centuries and have minimal side effects compared to traditional medications (Hassan et al., 2016; Hernández-Rodríguez et al., 2020; Bilal et al., 2021; Negi and Jain, 2022). Living organisms have a complex defense system against unstable free radicals that are constantly exposed to both internal and external factors. When free radicals accumulate in cells, they can cause oxidative stress and damage to the cells. However, the body’s normal metabolism produces antioxidant defense systems that continuously destroy free radicals and prevent cell damage (Muhammad et al., 2024). In recent years, free radicals and antioxidants have become increasingly important subjects of study. When the body’s metabolism is healthy, antioxidants and free radicals are in balance. Some argue that a decrease in antioxidant defense and an increase in oxidative stress and lipid peroxidation may play a role in the development of cancer (Başkol et al., 2007; Karabulut and Gülay, 2016; Aslankoç et al., 2019). Previously, the genus Allium was initially categorized within the Alliaceae family and then within the Liliaceae family in older categorization systems. Nevertheless, molecular phylogenetic research has demonstrated that the classification of the Liliaceae family is not monophyletic (Knud, 1998). As a result, the genus has been reclassified and is now considered part of the Amaryllidaceae family (previously known as the Alliaceae family) based on the APG III classification system (Chase et al., 2009). *Allium turcicum Özhatay & Cowley* is a member of the Amaryllidaceae family, which was identified as an endemic plant by N. Özhatay, and grows in high altitude, dry, stony areas around Batman (Sason), Siirt, and Halkis Mountain. This medicinal plant can grow without any maintenance during April and May. It is a native plant that offers various local benefits and is commonly consumed as food when mixed with rock salt and crushed (Jill et al., 1994). The purpose of this study was to investigate the potential medicinal benefits of the extract from *A. turcicum* Özhatay & Cowley on various cell lines. Specifically, we examined its cytotoxic and cell migration effects on HUVEC, HCT-116, and U373 cell lines, and measured its antioxidant activities. This is the first study to explore these effects of ATÖCE.

## Methods and materials

### Preparation of Allium turcicum Özhatay & Cowley Extract

**FIGURE 1:**
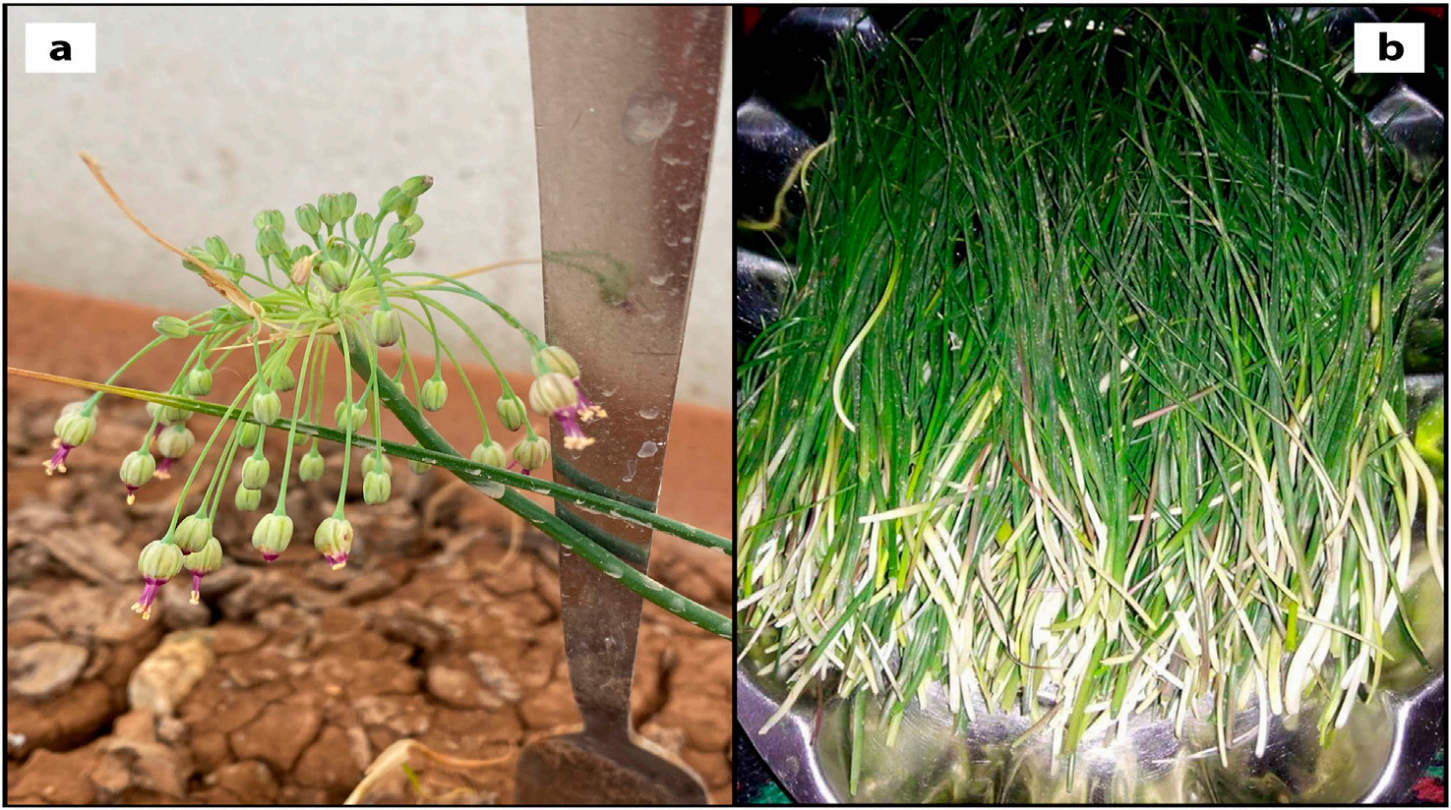
Morphological view of *Allium turcicum Özhatay & Cowley*; **(A)** after flowering **(B)** the collected parts before flowering.

The study material, *A. turcicum* Özhatay & Cowley endemic species, was collected from Batman-Sason Yakabağ Kaman region in April 2023 by Mehmet Fırat Baran and identified by Hülya Hoşgören. The samples dried according to herbarium techniques are kept in Mardin Artuklu (Cumali Keskin voucher no. MAU: 2023–26) and Dicle University Herbariums (Hülya Hoşgören, DUF 6995). To prepare *A. turcicum Özhatay & Cowley* (ATÖCE) methanolic extract, its plants, and roots were purchased from the public market in the Sason district of Batman province of Turkey in April 2023 (Figure 1). We washed the freshly consumed green parts with distilled water and dried them on blotting paper^12^. Next, we weighed 15 g of the dried plant and placed it in 100 mL of methanol (%99.6 purity) for a week. After that, we filtered the mixture and removed the methanol in the filtrate using a Heidolph 94,200 rotary evaporator at 70°C. Finally, we obtained the extract.

### Cell culture and cytotoxic activities of Allium turcicum Özhatay & Cowley via the MTT assay

Cytotoxic activity experiments on cells were conducted in the Cell Culture Laboratory of the Faculty of Veterinary Medicine at Dicle University. HCT-116 (Colorectal Carcinoma), U373 (Human glioblastoma astrocytoma) cancer cell lines, and healthy HUVEC (Human Umbilical Vein Endothelial Cells) cell lines were used in the study. Cell cultures were obtained commercially from the American Type Culture Collection (ATCC).

The cell lines were incubated in T75 culture flasks at 37°C in a 5% CO_2_ medium. The cells were treated with DMEM (Gibco 41965039, England) medium containing 10% FBS, 100 U/mL of penicillin, and 100 U/mL of streptomycin.

When the cells reached 80%–90% confluency, they were removed from the flasks and counted using the hemocytometric method. For each time application of 24 and 48 h, HUVEC, HCT-118, and U373 cells were seeded into 96-well plates in three replications with 7.5 × 10^3^ cells for HUVEC and 5 × 10^3^ cells for HCT-116 and U373 in each well with 90 µL medium (Muhammad et al., 2021; Muhammad et al., 2023). Subsequently, ATÖCE was applied at varying concentrations (2,000, 1,000, 500, 250, 125, 62.50, and 31.25 μg/mL), and the control group was given ultrapure water. We used a UV/Vis MTT test to assess changes in cell viability at 24 and 48 h’ post-application. 10 μL of MTT solution (5 mg/mL) was added to each well. The cells were then incubated for 3 h at 37°C in a humid atmosphere with 5% CO_2_. After 3 h, the medium in the wells was removed and replaced with 100 µL of DMSO. Once the plates had been left in the shaker for 20 min at room temperature, each well’s optical density (OD) values were measured at 540 nm using a UV/Vis Spectrophotometer (Multi Scan Go, Thermo) (Kandemir and Ipek, 2023).

The survival rate was calculated using as follows:

Survival Rate = ABS of Treatment/ABS of Control × 100% (Majeed et al., 2019; Wang et al., 2023).

### Cell migration assay

To conduct the study, the cell lines were planted in 6-well plates. Once the cells reached 80% confluency, a straight line was drawn from 12o to 6o’clock using a sterile pipette tip, creating a wound between the cells. The wells were washed twice with PBS to remove any dead cell residues. The treatment groups were given a medium containing ATÖCE equal to the IC50 values, while the control groups only received a medium. The degree of wound closures and speeds were compared between the groups to assess the cells’ migration toward the wound area. Images were taken at 24 and 48 h with an inverted microscope and evaluated comparatively (Zhao et al., 2015).

The migratory area was calculated by the formula:

Migratory Area (%) = (Area [0 day]-Area [24 h/48 h])/Area [0 day] × 100% (Wang et al., 2023).

### Determination of DPPH free radical removal activity

Determining antioxidant activity in all the prepared samples using a method developed by Wu et al. (2006). This method reduces a stable free radical known as 2,2-diphenyl-1-picrylhydrazyl (DPPH) in the presence of antioxidant compounds that donate hydrogen atoms and electrons. The result is a characteristic purple color that is measured spectrophotometrically. At first, 4 mL of 0.0004% (w/v) methanolic DPPH solution was mixed with 1 mL (0.2–1.0 mg) of extract solutions. The mixture was then incubated for 30 min at room temperature in the dark before taking absorbance readings at 517 nm.

By using the absorbance values of the samples, the % inhibition value was calculated by the formula:

$$\%\text{I}\text{n}\text{h}\text{i}\text{b}\text{i}\text{t}\text{a}\text{t}\text{i}\text{o}\text{n}=\frac{\text{A}\;\text{c}\text{o}\text{n}\text{t}\text{r}\text{o}\text{l}-\text{A}\;\text{s}\text{a}\text{m}\text{p}\text{l}\text{e}}{\text{A}\;\text{c}\text{o}\text{n}\text{t}\text{r}\text{o}\text{l}\times 100}$$

The inhibition values that were obtained have been plotted against the extract concentrations, which were determined in mg/mL. The concentrations of each extract that caused a 50% color opening were then calculated as the IC_50_ value. BHA was utilized as a positive control.

### Determination of ABTS free radical removal activity

ABTS free radical scavenging activity test was performed according to the method of Re et al. (1999). First of all, ABTS and potassium peroxodisulphate solutions were mixed and kept at room temperature for 12–18 h in a darkened environment. Before starting the experiment, it was diluted with ethanol at a wavelength of 734 nm in the spectrophotometer until the 0.700 absorbance value was read. Then the ABTS solution prepared with the samples was mixed and incubated at room temperature for 30 min. After this period, the absorbance values of the samples were measured at 734 nm. BHA solutions prepared at 5 different concentrations were used as the positive control. After the percent values of antioxidant activities were found, the corresponding IC_50_ values were calculated.

### Total phenolic content measurement

Folin-Ciocalteu reagent (Folin Phenol Reagent or Folin-Denis reagent) is a mixture of phosphomolybdate and phosphotungstate reagent, which has been used in the colorimetric determination of phenolic and polyphenolic antioxidants. Phenolic compounds formed a colored complex with Folin-Ciocaltaeu reagent in an alkaline medium, and the maximum absorbance of the constructed purple-violet complex was measured at 700 nm after 2 h of incubation. The obtained values were calculated using the gallic acid equivalent (mg GAE/g extract) equation (Singleton and Rossi, 1965). Gallic acid was prepared at five different concentrations for the calibration chart required to calculate gallic acid equivalence.

### CUPRAC (Copper (II) ion reducing capacity) test

The CUPRAC experiment was performed according to the method by Apak et al. (2006). The prepared CUPRAC solution was placed in the microplate wells, and 30 µL of the 1 mg/mL extract solution dissolved in its solvent was added. It was read at 450 nm after incubation at room temperature for 30 min. Obtained absorbance values were calculated in the trolox equivalent (mg TE/g extract) equation. Trolox was prepared in 4 different concentrations for the calibration chart required to calculate the trolox equivalence (Apak et al., 2006).

### Metal chelating experiment

The experiment for determining the metal chelating capacity was carried out according to Dinis et al. (Dinis et al., 1994; Hassan et al., 2021) The plant extracts were dissolved in their solvent, and the positive control EDTA was dissolved in methanol at 1 mg/mL 3.2 mL of distilled water was added to the extract solutions, and firstly, 100 µL of FeCl_2_ and then 200 µL of ferrozine were added. It was mixed well and incubated for 10 min at room temperature. Afterward, measurements were made at 562 nm in the spectrophotometer. The extracted data’s percent antioxidant activity values were calculated by substituting them in the equation.

### Statistical analysis

The experiments were conducted three times on separate days for accuracy in your study. After obtaining the results, the GraphPad Prism 8 program was used to calculate ATÖCE’s inhibitory concentration (IC_50_) value. The image-j program was utilized to analyze wound healing and express it as a percentage. The data was analyzed using the IBM SPSS 21.0 package program, with a statistical significance level of *p* < 0.05 being accepted (Chen et al., 2013; Hande et al., 2022; Kandemir and Ipek, 2023).

## Results and discussion

### Cytotoxic effects of ATÖCE

*Allium turcicum Özhatay&Cowley* plant is an endemic plant frequently consumed as a food product before flowering, with the thought of having various benefits in the area where it grows. However, no studies address the consumption dose limitation or report its cytotoxic effects.

This study aimed to evaluate the cytotoxic, cell migration, and antioxidant effects of ATÖCE in HCT-116 and U373 cancer cell lines and HUVEC healthy cell lines to look for hope in cancer treatment. Assessing cell viability can be best achieved through MTT procedure. This process relies on the activity of the mitochondria, which breaks down Tetrazolium bromide salt in living cells, resulting in the formation of purple formazan crystals (Malmir et al., 2020).

**FIGURE 2:**
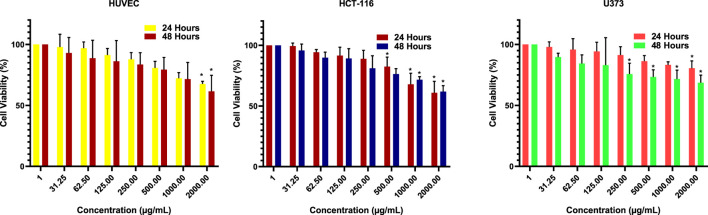
Cell viability percentages of ATÖCE (24 and 48 h). The effect of ATÖCE was concentration and time-dependent in both cell lines. *P < 0.05 compared to their control groups.

The cytotoxic activities of ATÖCE on HCT-116, U373 cancer cell lines, and healthy HUVEC cell lines were analyzed by the MTT method. Data on the activities of ATÖCE on HCT-116, U373, and HUVEC cell lines according to the MTT assay result are presented in Table 1 and Figure 2. We used ATÖCE on the cell lines in our study and observed that it can inhibit their growth. At varying time intervals and concentrations, we found that the death rate increased significantly in these cell lines.

**TABLE 1:** Cytotoxic effects of ATÖCE on cell lines (n = 3, x ± sx, 24 and 48 h).

	Cytotoxic effects of ATE on cell lines (n = 3, X ± sx, 24 h)							
		31.25 μg/mL	62.5 μg/mL	125 μg/mL	250 μg/mL	500 μg/mL	1,000 μg/mL	2,000 μg/mL
Cell Viability %	HUVEC	97.80 ± 10.50	96.90 ± 5.20	91.30 ± 5.40	87.80 ± 5.50	80.80 ± 5.30	72.30 ± 4.60	67.60 ± 2.10
	HCT-116	99.40 ± 02.40	94.30 ± 2.30	91.50 ± 6.90	88.80 ± 7.00	82.50 ± 7.80	67.90 ± 9.20	60.80 ± 9.50
	U373	98.00 ± 04.10	95.80 ± 8.90	94.30 ± 7.50	91.30 ± 6.80	86.30 ± 4.60	83.30 ± 2.50	80.40 ± 6.10

	Cytotoxic effects of ATE on cell lines (n = 3, X ± Sx, 48 Hours)							
		31.25 μg/mL	62.5 μg/mL	125 μg/mL	250 μg/mL	500 μg/mL	1,000 μg/mL	2,000 μg/mL
Cell Viability %	HUVEC	93.00 ± 12.70	88.60 ± 14.80	86.20 ± 17.00	83.50 ± 09.70	79.30 ± 10.00	71.60 ± 13.70	61.50 ± 13.10
	HCT-116	95.80 ± 05.20	89.80 ± 04.50	89.20 ± 08.10	81.00 ± 10.40	76.30 ± 04.40	71.60 ± 2.50	61.80 ± 04.90
	U373	94.60 ± 01.60	86.90 ± 04.70	78.80 ± 10.60	75.80 ± 09.30	73.30 ± 05.60	71.60 ± 7.20	68.40 ± 06.20

**FIGURE 3:**
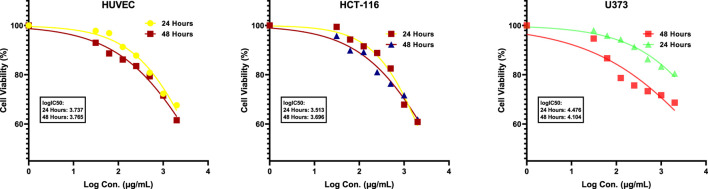
logIC_50_ of ATÖCE (24 and 48 h).

Based on the data gathered, when the highest concentration was administered to the HCT-116 cell line, it exhibited a 60.80% cytotoxic effect at 24 h and a 61.80% cytotoxic effect at 48 h. For the U373 cell line, the percentage was 80.40% at 24 h and 68.40% at 48 h. As for the HUVEC cell line, our healthy cell line, the ratio was 67.60% at 24 h and 61.50% at 48 h. Additionally, the logIC_50_ values after 24 and 48 h of application were determined as 3.737, 3.765 μg/mL; 3.513, 3.696 μg/mL; 4.476 and 4.104 μg/mL for HUVEC, HCT-116, and U373 cells, respectively (Figure 3). It is commonly known that cytotoxic and antitumor agents do not discriminate and may harm healthy cells that are actively multiplying. Therefore, there is a significant effort to explore new compounds that can more effectively target cancer cells while minimizing damage to healthy cells (Kocyigit et al., 2016). Colorectal cancer is a prevalent global disease (Xi and Pengfei, 2021). Regrettably, it is a prominent factor contributing to mortality in both males and females (Xi and Pengfei, 2021). The condition can only be treated by surgically excising the tumor (Xi and Pengfei, 2021). Despite advancements in treatment and early detection, almost 50% of individuals with this disease ultimately succumb to it (Issa and Noureddine, 2017). Chemotherapeutic medications can cause notable adverse effects, leading to an increased utilization of naturally sourced substances for treating cancer. Research has demonstrated that phytochemicals present in plants can serve as an alternative for cancer prevention and treatment (Afrin et al., 2020). It is also important to note that herbal products are not toxic to normal cells and are generally better tolerated (Huxley et al., 2009; Kocyigit et al., 2016; Cassiem and de Kock, 2019; Nelson et al., 2020). After reviewing the literature, it has been observed that various phytochemicals with anti-tumor properties have been tested on HCT-116 and U373 cancer cells. However, we could not locate any studies exploring the cytotoxic impact of ATÖCE on U373 and other cancer cell lines (Yokoyama et al., 2001; Abdullah Thani et al., 2012; Majeed et al., 2019; Nelson et al., 2020; Bilal et al., 2021). After analyzing the MTT results from our study, it was discovered that ATÖCE had the most potent cytotoxic effect on HCT-116 compared to other cell lines, depending on the dosage and duration. This effect was found to be statistically significant.

Our research found that ATÖCE had statistically significant cytotoxic effects on the U373 cell line. However, these effects were less pronounced than the other cell lines. This could be attributed to the high invasiveness of the U373 cell line.

### Cell migration assay

**FIGURE 4:**
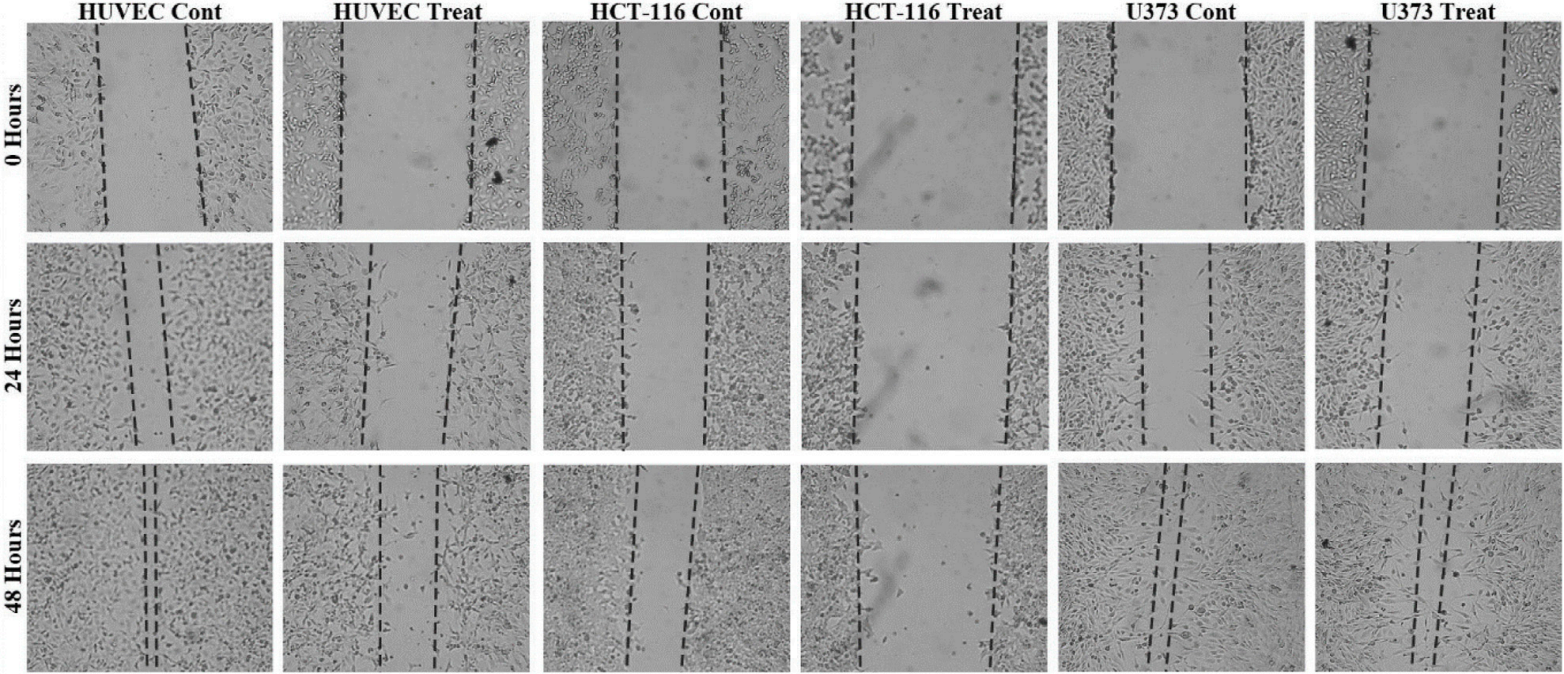
Antimigration effect of ATÖCE on healthy and cancer cell lines. We analyzed the effect of ATÖCE on the migration of HUVEC, HCT-116, and U373 cells by conducting a wound-healing assay. The cells were incubated with or without ATÖCE for 48 h and then observed under an inverted microscope at 24 and 48 h to see the cells that had migrated into the cavity. The black lines indicate the boundary of the blank area (Kocyigit et al., 2016).

A cell migration assay was conducted to assess the impact of ATÖCE application on cancer cell metastasis. After 48 h of the experiment, the cells treated with ATÖCE were compared to the untreated control cell groups. After analyzing our results, we found that treating cells with ATÖCE significantly affected their migration potential as measured by wound width. However, after 48 h, this effect was not statistically significant for U373 cells. We observed that treated cells had a slower wound closure rate than untreated control cells, suggesting that ATÖCE has an inhibitory effect on the cells’ migration potential, as shown in Figure 4.

Based on the data presented in Figure 4, it was observed that HUVEC, HCT116, and U373 cells showed migration after 24 h of observation. Compared to the control group, the migration percentages for these cells were 54.57%, 37.42%; 23.96%, 8.57%; and 47.04%, 26.41%, respectively. After 48 h, migration was still observed in the groups, with percentages of 94.74%, 59.49%; 45.24%, 26.79%; and 87.26%, 81.60%. Colorectal cancer (CRC) is a deadly cancer that affects people all over the world (Xi and Pengfei, 2021). Even though the diagnosis and treatment options have improved, the survival rate for this disease is less than 40% (Issa and Noureddine, 2017). The main reasons for the failure of cancer treatment and death are cancer cells’ abnormal movement, invasion of tissues, and metastasis. Cancer cells migrate from the primary tumor mass to nearby tissues during metastasis (Lin et al., 2022; Tarawneh et al., 2023).

Our study to investigate the possible effect of ATÖCE on the migration and invasion ability of cell lines showed us that ATÖCE has significant anti-CRC (anti-human colorectal cancer) activity, especially by significantly inhibiting the migration and invasion capacity of HCT-116.

In light of the results presented here, we can say that ATÖCE is a promising agent for colon cancer, with its effectiveness to be revealed by more comprehensive studies.

Glioblastoma multiforme (GBM) is a type of cancer that affects the brain and has the highest mortality rate among all brain cancers (Ostrom et al., 2013). One of the reasons why GBM is so aggressive and deadly is because the cancer cells can migrate within the brain, which promotes highly invasive cell growth and malignancy (Lattier et al., 2020). Unfortunately, this ability of cancer cells to migrate is also one of the main reasons GBM treatments often fail (Erdogan and Eroglu, 2019; Giannakopoulou et al., 2022; Oliva et al., 2022).

**FIGURE 5:**
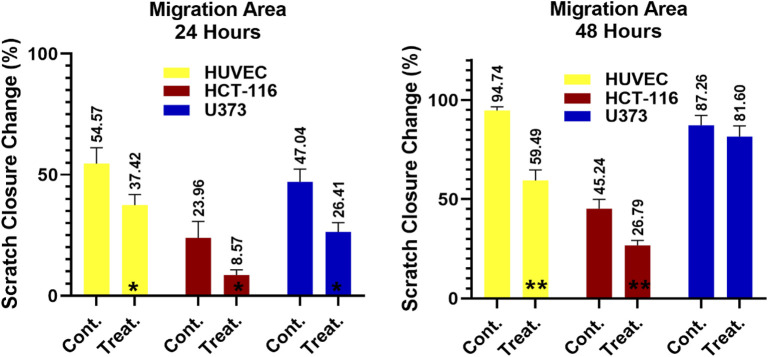
Scratch closure rate of ATÖCE on healthy and cancer cell lines at 24 and 48 h. After 24 and 48 h of incubation, there was a noticeable decrease in the migration of cells to the wound site (*p < 0.01, **p < 0.01 compared to their control groups).

In our research, we discovered that the effectiveness of ATÖCE in impeding cell migration on the U373 cell line is lower than that of other cell lines (Figure 5). This finding can be explained by the high level of invasiveness of the U373 cell line. Our study is the first to provide evidence that ATE can inhibit cell migration, suggesting that it could be a potential therapeutic agent for preventing the spread of cancer cells.

### DPPH free radical scavenging activity results

We calculated the extract of *A. turcicum Özhatay & Cowley* at main concentrations of 1 mg/mL, 2 mg/mL, 4 mg/mL and 8 mg/mL. From there, we created four intermediate concentrations with different ratios. After incubation, we measured the absorbances using a spectrophotometer and calculated the IC_50_ value. Table 2 shows the IC_50_ value for *A. turcicum Özhatay & Cowley* and the positive control BHA. A lower IC_50_ value indicates higher antioxidant activity.

**TABLE 2:** DPPH free radical scavenging activity results (IC50).

DPPH free radical scavenging activity (IC50) (mg/mL)	
Allium turcicum Özhatay & Cowley	1.90 ± 0.04
BHA	0.02 ± 0.01

The study showed that as the concentrations of plant extracts increased, there was also an increase in the scavenging of DPPH free radicals (as shown in Table 3). Although the results obtained were lower when compared with BHA at the determined concentrations (Table 4), a regular increase in concentration-dependent biological activity was observed. The activity differences between plants may be due to the different types and densities of the compounds they contain. In a study conducted with the blackberry plant (*Syzygium cumini* L.), different antioxidant activity values were measured even in samples from other parts of the plant (Halim et al., 2022; Shahar et al., 2023). Yildiztekin et al., in their study to determine the antioxidant activities of *Crocus mathewii*, reported that the ethyl acetate fraction of the shallot and aerial parts of the plant showed higher activity than the BHA standard. The IC_50_ value of the aerial parts’ DPPH radical scavenging activity was 36.21 ± 0.76 mg/L, while the IC50 value of the onions was calculated as 33.87 ± 0.02 mg/L. The IC50 value of the BHA standard was determined as 57.31 ± 0.25 mg/L (Yildiztekin et al., 2016; Muhammad et al., 2021).

**TABLE 3:** DPPH free radical scavenging activity results (%).

DPPH free radical scavenging activity results (%)				
	1.0 mg/mL	2.0 mg/mL	4.0 mg/mL	8.0 mg/mL
Allium turcicum Özhatay & Cowley	26.90 ± 0.80	59.30 ± 0.20	84.70 ± 0.60	91.50 ± 0.70

**TABLE 4:** DPPH free radical scavenging activity results (%).

DPPH free radical scavenging activity results (%)				
	0.01 mg/mL	0.02 mg/mL	0.03 mg/mL	0.04 mg/mL
BHA	34.80 ± 1,90	61.40 ± 2,90	75.50 ± 1,40	83.10 ± 1.00

### ABTS free radical scavenging activity results

To determine the activity of *A. turcicum Özhatay & Cowley* extract, 1 mg/mL, 2 mg/mL, 3 mg/mL, 4 mg/mL and 5 mg/mL main concentrations were calculated. Five intermediate concentrations at different ratios were created from the main stock, and the IC_50_ value of the absorbances obtained in the spectrophotometer at the end of the incubation period was calculated. According to the calculations, the IC_50_ value of the *A. turcicum Özhatay & Cowley* plant is given in Table 5, together with the BHA used as a positive control. The closer the IC_50_ value is to the BHA values (Table 6), which is the positive control, the higher the free radical scavenging activity of the plant. Due to the increase in concentration, the values of plant extracts were observed simultaneously with the rise in BHA values (Table 7).

**TABLE 5:** ABTS free radical scavenging activity results (IC_50_).

ABTS free radical scavenging activity results ((IC50) (mg/mL)	
A. turcicum	0.20 ± 0.10
BHA	0.01 ± 0.01

**TABLE 6:** ABTS free radical scavenging activity results (%).

ABTS free radical scavenging activity results (%)					
A.Turcicum	1.0 mg/mL	2.0 mg/mL	3.0 mg/mL	4.0 mg/mL	5.0 mg/mL
	53,90 ± 0,70	81,90 ± 0,20	87,00 ± 0,40	90,20 ± 0,70	93,70 ± 0,60

**TABLE 7:** ABTS free radical scavenging activity results (%).

ABTS free radical removal activity results (%)					
BHA	0.01 mg/mL	0.02 mg/mL	0.03 mg/mL	0.04 mg/mL	0.05 mg/mL
	38.30 ± 3.40	66.54 ± 3.80	92.20 ± 1.70	97.10 ± 0.60	98.20 ± 0.50

In the study with the leaf extract of *Dalbergia sissoo*, the percent inhibition of ABTS radical was 83.21% ± 1.41% at the highest concentration tested; In the study conducted with the leaf extract of *Bauhinia variegata*, it was reported to be 89.06% ± 0.34%. They showed activities close to ascorbic acid used as a standard at the highest concentration (Bakshi et al., 2022).

### Total phenolic content results

**FIGURE 6:**
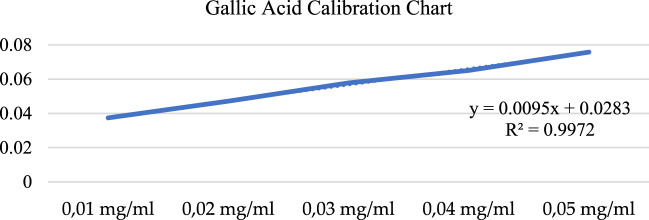
Gallic acid calibration chart.

A calibration chart was obtained using five different concentrations of gallic acid (Figure 6), and the gallic acid equivalence (mg GAE/g) of plant extracts was calculated using this chart.

The total phenolic content of the above-ground methanol extract of *A. turcicum Özhatay & Cowley* at a concentration of 1 mg/mL was found to be 0.26 ± 0.044 mg GAE/g (Table 8). Datta et al.^45^ reported the phenolic content of methanol extract as 127.58 ± 0.45 mg GAE/g in their study with *Cleome rutidosprema*, one of the underutilized plants of India. In the same study, the phenolic content of the methanol extract of the *Aluda mutica* plant was found to be 66.54 ± 2.78 mg GAE/g (Datta et al., 2022). One of the research reported the phenolic content of hydro methanolic extracts of two endemic plants from Turkey (*Nepeta italica* subsp. *Cadmea* and *Teucrium sandrasicum*) as 72.5 ± 1.59 mgGAEs/g and 150.18 ± 2.73 mgGAEs/g, respectively (Kaska et al., 2019).

**TABLE 8:** Results of total phenolic substance content.

Total phenolic substance (mg GAE/g)	
Allium turcicum Özhatay & Cowley	0.30 ± 0.01

### CUPRAC (Copper (II)) ion reducing capacity

**FIGURE 7:**
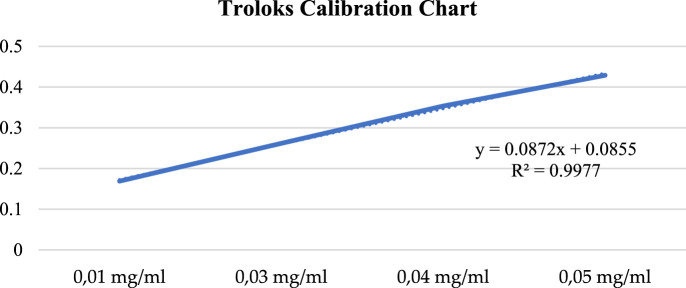
Troloks calibration chart.

A calibration chart was obtained using four different concentrations of trolox (Figure 7), and trolox equivalence (mg GAE/g) of plant extracts was calculated using this chart.

The copper (II) ion reducing capacity of the methanol extract of *A. turcicum Özhatay & Cowley* plant at 1 mg/mL concentration was found to be 0.28 ± 0.09 mg TE/g (Table 9).

**TABLE 9:** CUPRAC experiment results.

CUPRAC assay activities (mg TE/g)	
Allium turcicum Özhatay & Cowley	0.30 ± 0.10

In the study conducted with some plants grown in the Giresun region, CUPRAC-reducing power activities in methanol-water extracts were 0.878 ± 0.023 in chard beet (*Beta vulgaris* L.); It was calculated as 0.823 ± 0.0941 in the mendek (*Aegopodium podagraria* L.) and 0.754 ± 0.022 mg TE/g in beet (*Capsella bursa-pastoris* L.). They stated that the reducing power increased with the increase in concentration.

### Metal chelation experiment results

The value of the *A. turcicum Özhatay & Cowley* plant methanol extract at a concentration of 0.1 mg/mL in terms of metal-chelating antioxidant activity was found to be 13.95 ± 1.148. This value is lower than the synthetic antioxidant EDTA (66.24% ± 1.41) (Table 10). Iron is one of the elements necessary for organisms; It causes free radical formation due to undesired oxidative reactions with components such as lipids and proteins. For this reason, the iron-reducing power of antioxidant substances is crucial. The study conducted for antioxidant activity with the Merzifon black grape variety (Vitis vinifera L.) determined the metal chelating capacity closest to the EDTA standard as 37.12 in the whole grape at room temperature (Gülhan et al., 2023). Takcı et al. found the metal chelating activity of *Rheum ribes* plant methanol extracts to be 49.70% ± 3.10%, which shows higher metal chelation activity than our results (Takci et al., 2021).

**TABLE 10:** Metal chelation experiment results (%).

Metal chelating experiment activities (0.1 mg/mL)	
EDTA	66.20 ± 1.40
Allium turcicum Özhatay & Cowley	14.00 ± 1.20

## Conclusions

Our study is the first original to evaluate ATÖCE’s effects on glioblastoma and colon cancer cells. The ATÖCE administered showed the ability to hinder the growth and movement of HUVEC, HCT-116, and U373 cells. Its cytotoxic effects varied according to concentration and duration of exposure. However, ATÖCE also exhibited toxicity to healthy HUVEC cells. Our analysis revealed that ATÖCE has high antioxidant activity. In conclusion, this plant, known for its medicinal properties, can serve as a supportive treatment, particularly for colon cancer.

Further investigation is required to fully determine the effectiveness of ATÖCE in the context of colon cancer management. Despite this, the initial results indicate that ATÖCE holds great promise as a prognostic modality for patients with this condition. Given the considerable morbidity and mortality associated with colon cancer, the development of novel and reliable prognostic tools is of paramount importance. The use of ATE to predict patient outcomes could have significant implications for clinical decision-making and patient care. Therefore, additional studies are necessary to validate the results and establish the role of ATÖCE in the management of colon cancer.

## Data Availability

The original contributions presented in the study are included in the article/supplementary material, further inquiries can be directed to the corresponding authors.
